# CRISPR-dCas9 Activation of TSG-6 in MSCs Modulates the Cargo of MSC-Derived Extracellular Vesicles and Attenuates Inflammatory Responses in Human Intervertebral Disc Cells In Vitro

**DOI:** 10.1007/s12195-025-00843-4

**Published:** 2025-02-05

**Authors:** Iker Martinez-Zalbidea, Gabbie Wagner, Nea Bergendahl, Addisu Mesfin, Varun Puvanesarajah, Wolfgang Hitzl, Stefan Schulze, Karin Wuertz-Kozak

**Affiliations:** 1 Department of Biomedical Engineering, Rochester Institute of Technology (RIT), Rochester, NY USA; 2 Medstar Orthopaedic Institute, Georgetown University School of Medicine Washington, Washington, DC USA; 3 Department of Orthopedics and Rehabilitation, University of Rochester Medical Center, Rochester, NY USA; 4 Research and Innovation Management (RIM), Paracelsus Medical University, Salzburg, Austria; 5 Department of Ophthalmology and Optometry, Paracelsus Medical University, Salzburg, Austria; 6 Research Program Experimental Ophthalmology and Glaucoma Research, Paracelsus Medical University, Salzburg, Austria; 7 Thomas H. Gosnell School of Life Sciences, Rochester Institute of Technology, Rochester, NY USA; 8 Schön Clinic Munich Harlaching, Spine Center, Academic Teaching Hospital and Spine Research Institute of the Paracelsus Medical University Salzburg (Austria), Munich, Germany

**Keywords:** Exosomes, Genomic engineering, Transcriptomic modulation, IVD, Regeneration, Proteomics

## Abstract

**Purpose:**

The purpose of this study was to boost the therapeutic effect of mesenchymal stem cell (MSC)-derived extracellular vesicles (EVs) by overexpressing the gene TSG-6 through CRISPR activation, and assess the biological activity of EVs from these modified MSCs *in vitro* on human intervertebral disc (IVD) cells.

**Methods:**

An immortalized human MSC line was transduced with a CRISPR activation lentivirus system targeting TSG-6. MSC-EVs were harvested by ultracentrifugation and particle number/size distribution was determined by nanoparticle tracking analysis. The efficiency of transduction activation was assessed by analyzing gene and protein expression. EV proteomic contents were analyzed by mass spectrometry. Human IVD cells from patients undergoing spinal surgery were isolated, expanded, exposed to IL-1β pre-stimulation and co-treated with MSC-EVs.

**Results:**

MSC-EVs presented size distribution, morphology, and molecular markers consistent with common EV characteristics. The expression level of TSG-6 was significantly higher (> 800 fold) in transduced MSCs relative to controls. Protein analysis of MSCs and EVs showed higher protein expression of TSG-6 in CRISPR activated samples than controls. Proteomics of EVs identified 35 proteins (including TSG-6) that were differentially expressed in TSG-6 activated EVs vs control EVs. EV co-Treatment of IL-1β pre-Stimulated IVD cells resulted in a significant downregulation of IL-8 and COX-2.

**Conclusions:**

We successfully generated an MSC line overexpressing TSG-6. Furthermore, we show that EVs isolated from these modified MSCs have the potential to attenuate the pro-inflammatory gene expression in IVD cells. This genomic engineering approach hence holds promise for boosting the therapeutic effects of EVs.

**Supplementary Information:**

The online version contains supplementary material available at 10.1007/s12195-025-00843-4.

## Introduction

Intervertebral disc (IVD) degeneration is one of the main contributors to low back pain, the leading cause of disability worldwide, with a lifetime prevalence of 80% of the populace worldwide and an estimated cost of up to 100 billion dollars in the US alone [1, 2]. Although IVD degeneration is commonly asymptomatic, it can be associated with pain, a condition often referred to as degenerative disc disease (DDD) [3–5]. One of the early hallmarks of DDD is inflammation of the disc, in which cells of the IVD release pro-inflammatory cytokines [3–8]. The continued secretion of pro-inflammatory cytokines leads to the overexpression of matrix-degrading enzymes, such as collagenases and aggrecanases (including MMP3, MMP13, and ADAMTS-4) [8]. A reduced synthesis of ECM components, in combination with increased release of aggrecanases and collagenases, causes a catabolic shift in the IVD, with a loss of ECM, and structural failure allows for the invasion of immune cells (which contribute to inflammation) and the innervation of nociceptive nerve fibers in the IVD, leading to pain [8–14].

Although cell therapy has been explored as a novel therapy for IVD degeneration, the microenvironment of the degenerated IVD is hostile for cell survival and engraftment, which can limit its efficacy [13, 15]. Mesenchymal stem cells (MSCs) exert their therapeutic effect through several different mechanisms. Currently, a major mechanism of action behind the therapeutic effect of MSCs is attributed to their paracrine effect, i.e. the secretion of soluble immunomodulatory, anti-apoptotic, and anti-oxidative factors [16]. Another similar route of action is through the secretion of extracellular vesicles (EVs), which have shown anti-inflammatory and tissue-protective effects in different tissues [17–20].

EVs are lipid-bound particles secreted by all types of cells in both, eukaryotic and prokaryotic organisms [21, 22]. They can be isolated from bodily fluids as well as conditioned medium [21]. EVs are often classified as exosomes, microvesicles or apoptotic bodies according to their biogenesis pathways, molecular markers, function, and size [21, 22]. However, the Minimal Information for Studies of Extracellular Vesicles (MISEV) guidelines recommend collectively referring to them by the generic term “Extracellular Vesicles” or “EVs” [21].

EVs can be taken up by other cells and are crucial for cell-to-cell communication, acting as nanocarriers for bioactive molecules, including proteins, lipids, and nucleic acids (including DNA, mRNA, miRNA, and lncRNA) [21, 23]. The effect of EVs can vary greatly depending on the “donor” and “host” cells, the location within the organism or physiological context, and can range from therapeutic to pathologic [21, 24–26].

EVs derived from a wide range of sources—including those derived from MSCs—have shown diverse therapeutic effects, spanning from immune regulation to promotion of tissue repair and cell survival [18–20, 24, 27–29]. For example, MSC-EVs can inhibit inflammation and promote proliferation of chondrocytes *in vitro* and in animal studies, indicating their potential for the treatment of osteoarthritis [19]. Similar tissue repair effects have been observed in skin and spinal cord injury models using MSC-EVs [30, 31]. Studies on nucleus pulposus (NP) cells in the IVD have shown that MSC-EVs promote cell survival and proliferation, reduce ECM catalysis and overall ameliorate IVD degeneration [27, 28, 32, 33]. MSC-EVs exert their therapeutic function through several mechanisms. MSC-EVs inhibit the NF-κB pathway, thereby reducing the release of pro-inflammatory and catabolic factors, and promoting cell survival and proliferation in chondrocytes [34, 35]. Micro RNAs (miRNAs) delivered by MSC-EVs contribute to their therapeutic function. For example, it has been suggested that MSC-EV-derived miR-21 can promote cell survival in NP cells and reduce IVD degeneration, while miR155-5p can promote cartilage regeneration in osteoarthritis [36, 37]. Although MSC-EVs have shown a clear therapeutic potential, their clinical translation is challenging due to a combination of effects, such as limited therapeutic effect, donor-to-donor variability, and EV heterogeneity [24, 26, 38]. As such, it is warranted to explore ways to enhance their therapeutic potential, for example through genomic engineering.

CRISPR-Cas9 technology is a highly versatile gene editing technology that has become one of the most useful tools in modern biomedical research. Variants of the CRISPR-Cas9 technology can be used to alter the gene expression of a gene of interest (GOI) without altering the DNA sequence [39–42]. Catalytically “dead” Cas9 (dCas9) lacks endonuclease function but can still be directed to target DNA with the appropriate single guide RNA (sgRNA), and by fusing dCas9 to a transcription activator or a repressor, gene expression can be altered [39, 41, 43–45]. For example, CRISPR activation can be achieved by genetically fusing dCas9 to a VP64 transactivation domain, a strong transcription activator, and targeting the promotor regions of the GOI [41, 42, 45]. The first CRISPR activation systems could only achieve modest transcriptional activation, but subsequent designs were able to produce more robust upregulation of gene expression by incorporating multiple activation domains into the dCas9 complex [43, 44, 46]. One of the most interesting approaches combines MS2-binding loops in the sgRNA backbone to recruit two additional activation domains, p65, and HSF1, into the dCas9-VP64 complex, resulting in a stronger synergistic tool called the “synergistic activation mediator (SAM)” [43, 44]. Among the many potential applications of CRISPR activation, activation of “therapeutic” genes could be useful to enhance the effect of MSC-EVs. While MSC-derived EVs have shown promise for treating inflammatory and degenerative conditions, their therapeutic efficacy can be limited by factors such as donor variability, heterogeneity, and suboptimal potency. Merely increasing EV quantity in a clinical setting may not be effective due to scalability challenges, as well as limited regenerative effects of EVs. To address this, genome editing techniques such as CRISPR/dCas9 activation can fine-tune the EV cargo by enhancing the expression of ‘therapeutic’ genes without introducing full-length transgenes. This approach is novel in the field of EV therapeutics and aims to create more potent and consistent EV preparations. Altering EV cargo at the source, rather than increasing the dosage, may offer a more controlled and reproducible strategy to enhance therapeutic potential.

A promising candidate GOI to activate is the Tumor necrosis factor-stimulated gene 6 (TNFAIP6 or TSG-6). TSG-6 released from MSCs has been identified as a key mediator of the anti-inflammatory effects in several tissues [47, 48]. It acts as an autocrine factor that influences many properties of MSCs by affecting transcription factors, cytokine expression, and other key biological pathways and mechanisms [47–51]. Recent studies have suggested that TSG-6 plays an important role in the therapeutic effects of MSC-derived EVs [52–55]. We hypothesized that genetic overexpression of TSG-6 leads to increased TSG-6 levels in EVs, as well as other concomitant cargo changes, resulting in MSC-EVs with enhanced anti-inflammatory effects.

Therefore, the aim of this study was to perform an *in vitro* feasibility study for using CRISPR/Cas9 activation of a promising target gene (i.e., TSG-6) to promote the clinical potential of MSC-derived EVs.

## Materials and Methods

### Human MSC Culture

ASC52telo cells, an hTERT immortalized human adipose derived mesenchymal stem cell line (ATCC® SCRC-4000™), were seeded at 5,000 cells/cm^2^ in basal media (MEM Alpha, Cytiva, SH30024) supplemented with 10% fetal bovine serum (FBS, Cytiva, SH30396) and 1% antibiotic–antimycotic (anti-anti, Gibco, 15240062). The culture medium was refreshed every 2–3 days, and cells were passaged when they reached approximately 80% confluence. All standard culture practices were conducted using cells under 20 passages. ASC52telo cells will be referred to as MSCs from here on.

### Lentiviral Transduction and TSG-6 Activation

Genetic overexpression of TSG-6 was achieved by transducing MSCs with the “SAM CRISPR Activator Lentivirus” (CRISPR Custom Lentiviral Vector, Sigma-Aldrich) system, which includes three lentiviral vectors with appropriate antibiotic resistance genes for selection: “dCas9-VP64-Blasticidin SAM CRISPRa Helper Construct 1”, “MS2-P65-HSF1-Hygromycin SAM CRISPRa Helper Construct 2”, and “CRISPRa SAM U6-gRNA:EF1-Puro” with the single guide RNAs (sgRNA) targeting the promotor region of the gene of interest TNFAIP6 (TSG-6) or Non-targeting (negative) sgRNA controls. Three different sgRNAs targeting TSG-6 were designed by the manufacturer, based on previously established research [43, 44]. Lentivirus functional titers were provided by the manufacturer as transcriptional unit concentration (TU/ml), and were determined via Colony Formation Unit (CFU) assays. Prior to starting the transductions, a 7-day kill curve for each antibiotic was performed on the MSCs to establish their sensitivity to puromycin, blasticidin, and hygromycin. For transduction, MSCs were seeded in 12 well plates with 1 ml of standard cell culture medium (MEM Alpha supplemented with 10% FBS and 1% anti-anti) at a density of 10,000 cells per well. The next day, the cells were transduced for 24 h with the lentivirus of choice at a multiplicity of infection (MOI) of 0.7, as recommended by the manufacturer, in culture medium supplemented with 200 µg/mL protamine sulfate (TCI, P0675). The dosage required to achieve an MOI of 0.7 was calculated based on the functional viral titer (TU/ml) provided by the manufacturer.

$${\textit{Volume of Viral Particle}}\, \left( \upmu {\text{l}} \right) = 1000\,\upmu {\text{l}} \times \frac{\textit{Number of Cells} \times \textit{Desired MOI}}{\textit{Viral Titer}\,\left( {\text{TU/ml}}\right)}$$

24 hours post-transduction, the growth medium was replaced with selection medium containing the antibiotic dosages previously determined (10 µg/ml Blasticidin, AAJ61883-FPL, Alfa-Aesar; 100 µg/ml Hygromycin, 30-240-CR, Corning; 0.5 µg/ml Puromycin, MIR5940, Mirus Bio). The media was replaced every 2 days and the duration of selection was 4 days for Puromycin, and 7 days for Blasticidin and Hygromycin. The lentiviral transduction and selection process was carried out serially, first for the “dCas9-VP64-Blasticidin SAM CRISPRa Helper Construct 1”, and then for the “MS2-P65-HSF1-Hygromycin SAM CRISPRa Helper Construct 2”. After generating stocks of transduced cell lines, these cells were further transduced with the sgRNA constructs “CRISPRa SAM U6-gRNA:EF1-Puro” (using 3 different sgRNA constructs) or alternatively non-targeting negative controls (NTC). The efficiency of the genetic activation was determined by RT-qPCR and Western Blot.

### MSC Trilineage Differentiation

In order to validate that the MSCs retain their multipotent identity after CRISPR activation, a trilineage differentiation assay was performed. TSG-6 activated MSCs (passage 11–13) were cultured for two weeks with adipogenic, osteogenic, or chondrogenic differentiation media (Human Mesenchymal Stem Cell Functional Identification Kit, R&D, SC006) and were subsequently fixed and stained by immunocytochemistry following the manufacturer’s specifications (n = 1). All differentiation supplements were included with the kit.

For adipogenic differentiation, MSCs were seeded at a density of 2.1 × 10^4^ cells/cm^2^, and cultured in MEM alpha culture medium until they reached 100% confluence, as indicated by the manufacturer. After reaching confluence, cells were cultured in adipogenic differentiation medium (MEM alpha with Adipogenic Supplement) for 14 days, replacing media every 3–4 days. Osteogenic differentiation was performed by plating 4.2 × 10^3^ cells/cm^2^ and culturing them in basal medium until they reached 70% confluence. Afterwards cells were cultured with osteogenic differentiation medium (MEM alpha with Osteogenic Supplement) for 14 days, replacing media every 3–4 days. Chondrogenic differentiation was done by pellet culture. Briefly, 2.5 × 10^5^ MSCs were transferred into a 15 ml conical tube and centrifuged at 200×g for 5 min, the supernatant was discarded, MSCs were resuspended in 0.5 ml of DMEM basal medium, and centrifuged once more without replacing the medium. MSCs were maintained in culture with chondrogenic differentiation medium (DMEM with Chondrogenic Supplement) for 14 days, replacing media every 2–3 days without disturbing the pellet. Negative controls were cultured in basal medium. After 14 days of culture, the adipogenic, osteogenic, and chondrogenic differentiation as assessed by immunocytochemistry as described below.

### Immunocytochemistry/-Histochemistry

MSCs cultured under adipogenic or osteogenic conditions (and negative controls on basal media) were fixed with 4% paraformaldehyde (PFA, Thermo Fisher Scientific, J19943-K2) for 20 min and blocked with 1% BSA (Sigma-Aldrich, A2153-50G) for 5 min. Afterwards, MSCs were incubated in permeabilization and blocking medium containing 0.3% Triton X-100 (Fisher Scientific, ICN19485450), 1% BSA, and 10% donkey serum (Sigma-Aldrich, D9663) in PBS (Cytiva, SH30028) for 45 min. After blocking, MSCs were incubated with primary antibody solutions containing Mouse Anti-Human Osteocalcein Monoclonal Antibody or Goat Anti-Mouse FABP4 Antigen Affinity-purified Polyclonal Antibody) diluted at a concentration of 300 µl/ml in blocking medium overnight at 4 °C, and then incubated with secondary antibody Donkey Anti-Goat IgG H&L (Alexa Fluor® 555, Thermo Fisher Scientific) and Donkey Anti-Mouse IgG H&L (Alexa Fluor® 488, Thermo Fisher Scientific) in the dark for 1 h at room temperature. Nuclei were stained with DAPI Solution (Thermo Fisher Scientific). Chondrogenic pellets were fixed with 4% PFA for 1 h and embedded in cryoprotectant (Fisher Healthcare Tissue-Plus™ O.C.T. Compound, 23-730-571) Untreated Controls of chondrogenic differentiation, i.e. MSCs cultured with basal media, did not form pellets. Next, pellets were frozen in liquid nitrogen and cut in sections between 5 and 8 µm with a cryostat (Leica CM1520). Sections were blocked and permeabilized with blocking solution for 45 min, afterwards incubated with Goat Anti-Human Aggrecan Antigen Affinity-purified Polyclonal Antibody diluted at a concentration of 300 µl/ml in blocking medium overnight at 4 °C, and then incubated with Donkey Anti-Goat IgG H&L (Alexa Fluor® 555) secondary antibody as previously described. Images were taken on an Olympus IX-81 fluorescence microscope.

### RNA Extraction and Gene Expression Analysis (RT-qPCR)

MSCs were lysed and mRNA was extracted with the RNeasy Mini Kit (Qiagen, 74104) following the manufacturer’s recommendations. RNA was quantified with a Nanophotometer N50 instrument (Implen) and 1000 ng RNA was reverse transcribed into cDNA using the High-Capacity cDNA Reverse Transcription Kit with RNase Inhibitor (Thermo Fisher Scientific, 4374967). Gene expression was analyzed by qPCR with the TaqMan™ Fast Advanced Master Mix (Thermo Fisher Scientific, 4444963) and the appropriate Taqman™ Gene Expression Assay (Hs00427620_m1 TBP, Hs00174103_m1 IL-8, Hs00200180_m1 TNFAIP6, Hs01555410_m1 IL-1β, Hs00153133_m1 COX2, Thermo Fisher Scientific, 4331182) on a QuantStudio 3 instrument and software (Thermo Fisher Scientific). The results are depicted as 2^−∆∆Ct^ values relative to TBP and non-target controls (in the case of TSG-6 activation in MSCs, n = 3), or IL-1β stimulation controls (for IVD experiments, n = 5).

### Western Blot

In order to determine protein levels of MSCs, cell lysates were analyzed by Western Blot (n = 3). Briefly, cells were rinsed with ice-cold PBS and lysed on ice with RIPA Lysis and Extraction Buffer supplemented with 100× phosphatase and protease inhibitor cocktail, diluted to a final concentration of 1× in RIPA buffer (Thermo Fisher Scientific, 89901 and 78444). For EV protein analysis, the final pellet of EV collection (see below) was resuspended in 100 µl of protein lysis buffer (n = 3). Samples were centrifuged at 14,000×g for 15 min at 4 °C and protein content was quantified by a BCA assay (Thermo Fisher Scientific, 23225). 10 µg of protein was mixed with 4X Laemmli buffer containing 10% β-mercaptoethanol (Sigma Aldrich, 444203), heated (95 °C, 5 min), and then loaded onto a Mini-PROTEAN TGX 7.5% protein gel (Bio-Rad, 4568024) together with a protein ladder (Thermo Fisher Scientific, 26617), using SDS-PAGE running buffer. Electrophoresis was performed on a Mini-PROTEAN Tetra Cell (Bio-Rad) and blots were transferred onto a 0.2 µm polyvinylidene difluoride (PVDF) Transfer-Blot Turbo Transfer Pack membrane (Bio-Rad, 1704156) using a Trans-Blot turbo transfer system (Bio-Rad, 1704150). The membranes were subsequently blocked in filtered 5% non-fat milk (Research Products International, M17200-500.0) in Tris-buffered saline-Tween (TBS-T) for 1 h at room temperature. After washing in TBS-T (3 × 10 min), membranes were incubated with primary antibodies (TSG-6 Mouse monoclonal, R&D, 259820; Hsp70 Rabbit monoclonal, Abcam, EPR16892; β-Actin Rabbit monoclonal, Cell Signaling Technology, 8457L) diluted 1:1000 in 5% bovine serum albumin (Sigma-Aldrich) in TBS-T overnight at 4 °C. The next day, membranes were washed again in TBS-T (3 × 10 min) before incubation with secondary antibodies (Anti-rabbit IgG HRP-linked Antibody, Cell Signaling Technology, 7074S; Anti-Mouse IgG HRP- linked Antibody, Cell Signaling Technology, 7076P2) diluted 1:2000 in TBS-T for 1 h at room temperature. Proteins were detected with a chemiluminescence substrate (SuperSignal West Femto Maximum Sensitivity Substrate, Thermo Fisher Scientific, 34095) and imaged on the ODYSSEY XF Imaging System (LICOR).

### Collection of EVs

Prior to the harvest of EVs, MSCs (“TSG-6 activated” and “NT” controls) were cultured as previously described until reaching 80% confluence. Medium was aspirated and washed with PBS and replaced with serum free basal media. 48 h after, the conditioned medium (CM) from MSCs was collected. Immediately after, the CM was centrifuged first at 300×g for 5 min and then subsequently at 2000×g for 15 min at 4 °C to remove cellular debris and other large particles. The supernatant was transferred to ultracentrifuge tubes (OptiSeal, Beckman Coulter, 361625) and centrifuged first at 15,000g × 30 min at 4 °C (Optima MAX-XP Ultracentrifuge, MLA-50 rotor, Beckman Coulter). Then, supernatants were ultracentrifuged at 100,000g × 120 min at 4 °C (Optima MAX-XP Ultracentrifuge, MLA-50 rotor, Beckman Coulter). The resulting pellet was resuspended in 500 µl of PBS and stored at − 80 °C.

### Characterization of EVs

MSC derived EV suspensions were characterized for size-distribution, morphology, surface markers, and TSG-6 protein cargo. Particle concentration and size distribution was measured by nanoparticle tracking analysis (NTA). Briefly, MSC-EV pellets were diluted at 1:20 with sterile-filtered PBS and analyzed using a Nanosight 300 instrument as described by the manufacturer’s protocol. Three 30-second measurements were recorded per sample, and data was processed with the NanoSight NTA 3.4 software (n = 3). The EV morphology was assessed by transmission electron microscopy (TEM) at the Electron Microscopy Shared Resource Laboratory at the University of Rochester Medical Center (URMC) (n = 1). Briefly, 3 microliter of the EV suspension were placed in a 200 mesh carbon formvar coated copper grid (Electron Microscopy Science, Pennsylvania, USA) following glow discharge for 30 s at 30 mA and incubation for 30 s. Excess sample was blotted and grids were rinsed sequentially with molecular grade water and a 0.75% solution of uranyl formate. Grids were air dried and imaged on a Talos L120C TEM with a 16 MP CETA camera at 8500 and 22 K magnification using the TIA software program (Thermo Fisher Scientific, Massachusetts, USA). Marker characteristics of the isolated EVs were assessed with an immunoblotting antibody array kit (Exo-Check™ Exosome Antibody Array, SBI, EXORAY200B) pre-printed with antibodies specific for markers found enriched in EVs (CD63, EpCam, ANXA5, TSG101, GM130, FLOT-1, ICAM, ALIX, and CD81) (n = 1). EV protein samples were collected by resuspending the final pellet of ultracentrifugation in 100 µl protein lysis buffer, and protein contents were quantified by BCA. 50 µg of EV protein samples were prepared with lysis buffer and labelling reagent following the manufacturer’s instructions. The labelled EV lysate and blocking buffer mix was incubated with the antibody array membrane at 2–8 °C overnight on a shaker. After incubation, the membrane was washed and then incubated for 30 min with detection buffer. Membranes were developed with a chemiluminescence substrate (SuperSignal West Femto Maximum Sensitivity Substrate, Thermo Fisher Scientific, 34095) and imaged on an ODYSSEY XF Imaging System (LI-COR Biosciences). TSG-6 content in EVs was measured by WB as described earlier.

### Mass Spectrometric Analysis of EVs

To analyze the proteome content of MSC derived EVs, EV pellets from WT, NTC and TSG-6 MSCs were collected as described above and resuspended in 100 µl PBS (n = 3). Protein quantification and downstream analysis were conducted at the University of Rochester Medical Center (URMC) Mass Spectrometry Core Facility, following their established procedures [56]. Briefly, 25 µg of protein per sample were treated with dithiothreitol (2 mM) and incubated at 55 °C for 60 min. Next, samples were treated with 10 mM of iodoacetamide in the dark for 30 min at room temperature. The solution was then treated with phosphoric acid (1.2%) and six volumes of binding buffer (90% methanol, 100 mM Tetraethylammonium bromide, pH 7.55). Solutions were added to S-Trap microcolumns (Protifi) and washed three times with binding buffer, with centrifugation at 4000×g for 1 min before and in between washes. Proteins were digested overnight at 37 °C with trypsin. The next day, peptides were collected, freeze dried in a Speed Vac (Labconco) and resuspended in 0.1% trifluoroacetic acid before mass spectrometry analysis.

Peptides were loaded onto a 75 µm × 2 cm trap column (Thermo Fisher) before re-focusing on an IonOpticks Aurora Elite 75 um × 15 cm C18 column using a Vanquish Neo UHPLC (Thermo Fisher), connected to an Orbitrap Astral mass spectrometer (Thermo Fisher). A gradient of Solvent A (0.1% formic acid in water), and Solvent B (0.1% formic acid in 80% acetonitrile) was used to separate the peptides. The Orbitrap Astral was operated in data-independent acquisition (DIA) mode, with MS1 scans conducted in the Orbitrap at a resolution of 240,000 and a maximum injection time of 5 ms across a range of 380–980 m/z. DIA MS2 scans were carried out in the Astral mass analyzer with a maximum injection time of 3 ms, utilizing a variable windowing strategy, including 2 Da windows from 380 to 680 m/z, 4 Da windows from 680 to 800 m/z, and 8 Da windows from 800 to 980 m/z. HCD collision energy was maintained at 25%, and normalized AGC was set to 500%. Fragment ions were detected within a scan range of 150–2000 m/z, and the cycle time was 0.6 s. Raw data was processed with DIA-NN version 1.8.1 (available at https://github.com/vdemichev/DIA-NN) [57]. Data analysis was performed utilizing library-free analysis mode within DIA-NN. To construct the library, the human UniProt database, specifically the ‘one protein sequence per gene’ dataset (UP000005640_9606, 4/7/2021), was employed, with the inclusion of ‘deep learning-based spectra and RT prediction’. Parameters for precursor ion generation were set as follows: a maximum of 1 missed cleavage, up to 1 variable modification for Ox(M), peptide lengths ranging from 6 to 30, precursor charge within 2–4, precursor m/z ranging from 380 to 980, and fragment m/z ranging from 150 to 2000. Quantification settings were configured for ‘Robust LC (high precision)’ mode with RT-dependent normalization, enabled MBR, protein inferences set to ‘Genes’, and ‘Heuristic protein inference’ disabled. Mass tolerances for MS1 and MS2, as well as the scan window size, were automatically adjusted by the software. Precursors were subsequently filtered based on library precursor q-value (1%), library protein group q-value (1%), and a posterior error probability threshold of 50%. Protein quantification utilized the MaxLFQ algorithm implemented in the DIA-NN R package (https://github.com/vdemichev/diann-rpackage) and the quantification of peptides within each protein group was conducted using the DiannReportGenerator Package (https://github.com/URMC-MSRL/DiannReportGenerator) and ProteinReportr (https://github.com/urmc-msrl/ProteinReportr), while plots were generated with QuartoReport (https://github.com/URMC-MSRL/QuartoReport) [58].

### GO-Term and Protein Network Analysis

Proteins that showed significant differential abundances in comparative proteomics were subjected to GO-term enrichment analyses using GO-net (selecting the namespaces ‘biological processes’ and ‘molecular functions’) [59] as well as statistical overrepresentation tests using PANTHER pathways [60] and protein network analyses using STRING [61]. For all analyses, statistically more abundant proteins were analyzed separately from statistically less abundant proteins and all identified and quantified proteins were used as the background. A threshold of p < 0.05 was set for all employed tools.

### Human IVD Cell Isolation and Culture

Human degenerated IVD cells were isolated from disc tissue obtained from patients undergoing spinal surgery due to disc herniation or DDD, with informed patient consent (IRB Number: University of Rochester Medical Center STUDY00005200) [62]. Briefly, IVD tissue was surgically excised, diced with a scalpel, and enzymatically digested with a mix of 0.2% collagenase NB4 (Nordmark) and 0.3% dispase II (Sigma-Aldrich) in 1x Dulbecco’s Phosphate Buffered Saline (DPBS, Cytiva) with 5% antibiotic-antimycotic (anti-anti, Gibco) overnight in a humidified incubator (37 °C, 5% CO_2_). Isolated IVD cells were cultured up to passage 2 in standard cell culture medium (Dulbecco’s Modified Eagle’s Medium/Ham’s F-12 (DMEM/F-12 Cytiva, SH30023) supplemented with 1% antibiotic–antimycotic (anti-anti, Gibco, 15240062) and 10% fetal bovine serum (FBS, Cytiva, SH30396). An overview of the donor characteristics is included in Supplementary Table 1.

### Human IVD Cell IL-1β and EV Co-treatment

IVD cells in passage 2–3 were seeded in 6 well plates at 300,000 cells per well in 2 ml of cell culture medium (DMEM/F12, Cytiva, SH30023) supplemented with 10% fetal bovine serum (FBS, Cytiva, SH30396) and 1% antibiotic–antimycotic (anti-anti, Gibco) and incubated at 37 °C and 5% CO_2_ for 24 h to allow for cell attachment (n = 5). Medium was then switched to serum-free standard media. Two hours later, each well was exposed to different treatment conditions: “Untreated control” conditions were maintained with fresh serum-free media for the duration of the experiment. “IL-1β inflammatory control” conditions received 2.5 ng/ml recombinant human IL-1β (Peprotech, 200-01B). “EV control” conditions were treated with an EV suspension, at a dosage of 1000 particles per cell (i.e., 300 million particles for 300,000 IVD cells). Lastly, “IL-1β + EV co-treatment” conditions were treated with 2.5 ng/ml IL-1β plus EV suspension (1000 particle per IVD cell). RNA samples were collected 24 h after initiation of serum starvation (i.e., 18 h of EV treatment). Particle dosages from EV suspensions were determined previously by NTA. EV suspensions from TSG-6 Activated MSCs (TSG-6 EVs) and Non-target MSCs (NT-EVs) were used in parallel. Cells were maintained in serum-free media for the duration of the experiment. Table 1 outlines the experimental conditions.

**Table 1.** General experimental setup for EV treatment and IL-1β pro-inflammatory co-treatment of IVD cells

	Untreated control	IL-1β control	EV treatment	IL-1β + EV co-treatment
*Start of starvation*				
2 h post-starvation	Replace media	2.5 ng/ml IL-1β	Replace media	2.5 ng/ml IL-1β
4 h post-starvation	Replace media	2.5 ng/ml IL-1β	EV treatment	EV treatment (2.5 ng/ml IL-1β + EV)
24 h post-starvation	RNA collection			

Basal medium for all experimental conditions was serum free basal cell culture medium (DMEM/F12 with 1% antibiotic–antimycotic).

### Statistical Methods

Independent Student t-tests and one sample t-tests and corresponding bootstrap-t tests based on 3000 Monte Carlo simulations were used to test means. Whisker plots with standard errors were used to illustrate results. All reported tests are one-sided based on our underlying directional hypothesis (means were less than the control, i.e. μ < 1), and p-values < 0.05 were considered as statistically significant. While multiple groups were included (e.g., IL-1β, EV treatment, co-treatment), primary comparisons focused on specific pairwise contrasts (e.g., IL-1β alone vs. IL-1β + EV). All statistical analyses in this report were performed by use of STATISTICA 13 (Hill, T. & Lewicki, P. Statistics: Methods and Applications. StatSoft, Tulsa, OK), MATHEMATICA (Wolfram Research, Inc., Mathematica, Version 13, Champaign, IL, 2022) and PASW 29 (IBM SPSS Statistics for Windows, Version 29.0., Armonk, NY).

## Results

### CRISPRa-SAM Modified MSCs Retain Trilineage Differentiation

**Fig. 1:**
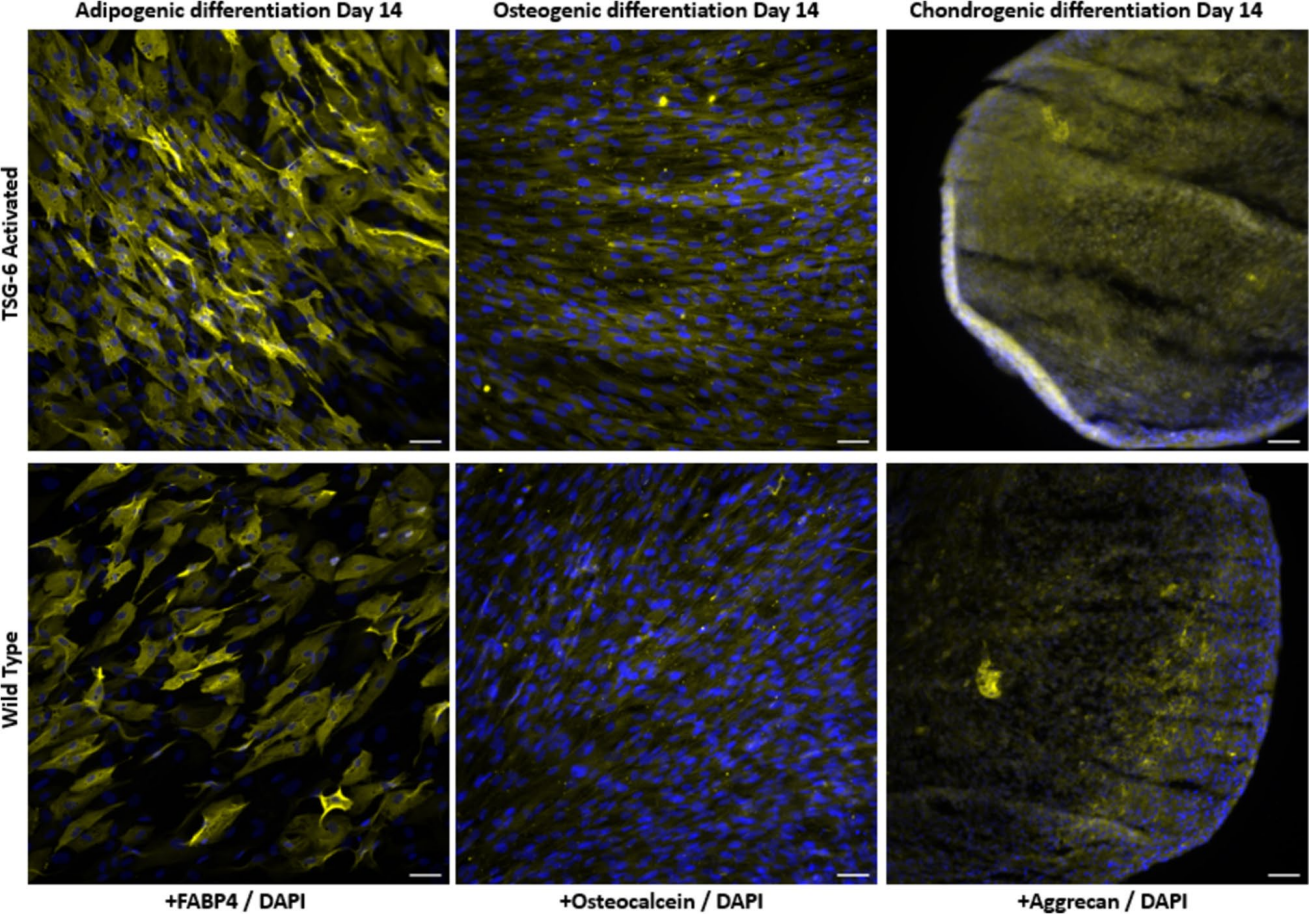
Representative immunofluorescence staining of trilineage differentiation markers: Adipogenic-FABP4 (Yellow), osteogenic–Osteocalcein (Yellow), and chondrogenic–Aggrecan (Yellow). Cell nuclei were stained with DAPI (blue). Scale bar 50 µm. (n = 1)

When TSG-6 activated MSCs and unmodified (wild type) controls (passages 15) were treated for 14 days under adipogenic, osteogenic and chondrogenic culture conditions, they both presented common trilineage differentiation markers (Fig. 1). Untreated controls (i.e. exposure to standard media) for each trilineage differentiation protocol were conducted in parallel for the duration of the experiment and lacked specific marker expression; untreated controls of pellet culture did not form chondrogenic pellets (Supplementary Figure 1).

As shown in Fig. 1, immunocytochemistry staining of adipogenic differentiation cultures of TSG-6 and WT MSCs showed strong expression of fatty acid binding protein 4 (FABP4), a common adipocyte marker. Similarly, osteogenic cultures presented positive staining for Osteocalcein, a factor secreted by osteoblasts. Lastly, chondrogenic pellets expressed aggrecan, a proteoglycan found in cartilage. Images suggest that TSG-6 activation did not visibly impair the trilineage differentiation potential of MSCs. These results suggest that CRISPR activated cells retain their trilineage differentiation capacity, a crucial characteristic of MSCs.

### Harvested EV Fraction Presents EV-like Characteristics

**Fig. 2:**
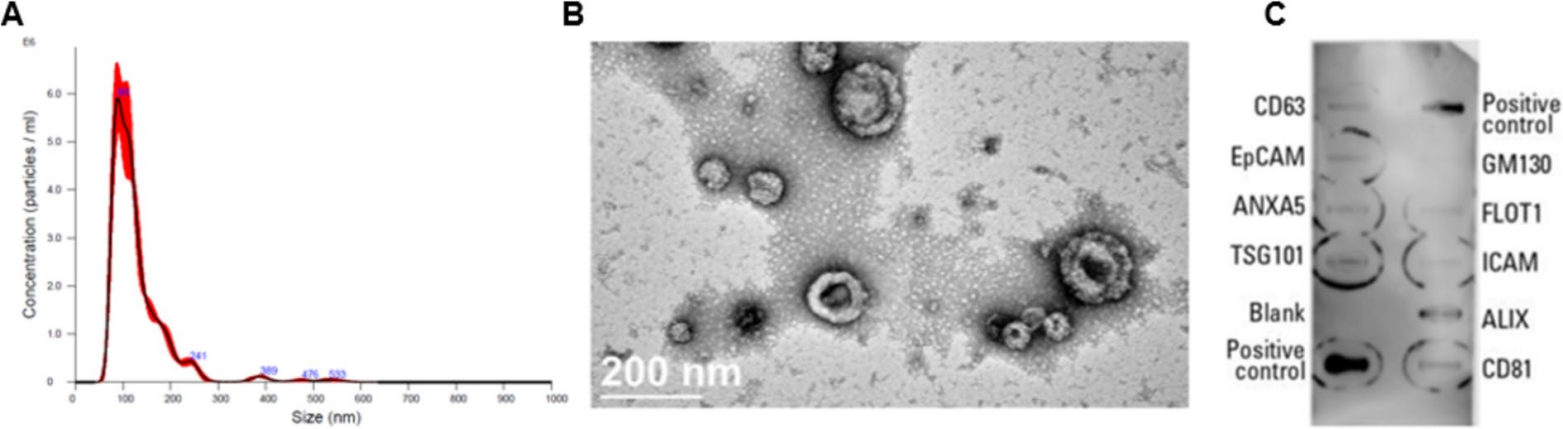
Size distribution, morphology and marker characterization of EVs from CRISPRa modified MSCs. **A** Concentration and size distribution of particles measured by NTA analysis 200 nm (mean of 130.3 ± 4.4 nm) (n = 3). **B** Representative TEM image of cup-shaped EV-like particles (Scale bar = 200 nm) (n = 1). **C** Antibody array showing the expression of multiple EV markers (n = 1). The “coffee-rings” seen in the antibody array are an artifact product of the film fabrication and do not affect the experiment

In order to assess the characteristics of the harvested EV fraction, we measured the particle-size distribution, morphology and expression of molecular markers. Nanoparticle tracking analysis revealed particle concentrations in ranges between 1 × 10^9^–1 × 10^10^ particles per ml, with the majority of the particles presenting a diameter below 300 nm (Fig. 2A). EV suspensions were imaged with TEM, and cup-shaped structures with diameters below 200 nm were observed (Fig. 2B). These results are consistent with particles that are broadly reported in the literature as “Exosome-like” or “EV-like” [21].

To further validate the identity of the isolated particles, the presence of protein markers was assessed with the antibody array Exo-Check™ Exosome Antibody Array (Fig. 2C). The array includes the standard markers, such as TSG101, CD and Calnexin: TSG101, a cytosolic protein found enriched in EVs was detected both in EV and MSC samples. Tetraspanin CD9 was also detected in EV samples, however much fainter than in MSC samples. Calnexin, an intracellular protein considered to be an “EV-depleted” marker, was only detected in MSC samples. In addition, the array measures expression of a wide range of other commonly used protein markers. We were able to verify the presence of several “EV-enriched” markers, including markers ALIX, FLO1 and tetraspanins CD63 and CD81. GM130, a Golgi apparatus marker (which is considered to be “EV-depleted” marker) was accordingly not detected. These results suggest that our sample contains a substantial concentration of EVs.

### CRISPR Activation Leads to Changes in Cell and EV Contents

**Fig. 3:**
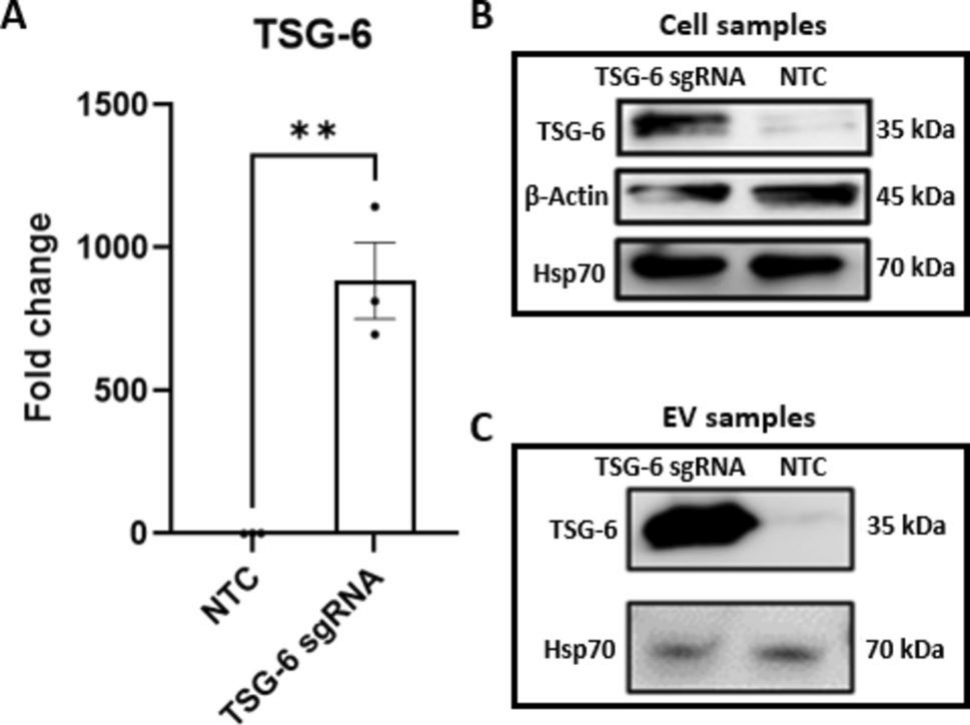
Gene and protein expression of TSG-6. **A** Gene expression of TSG-6 in transduced MSCs (TSG-6 sgRNA) and Non-target controls (NTC) (n = 3). **B** Protein expression of TSG-6, β-Actin, and Hsp70 in transduced MSCs (TSG-6 sgRNA) and Non-target controls (NTC) (Representative image, n = 3). **C** Protein expression of TSG-6, and Hsp70 in EV samples harvest from transduced MSCs (TSG-6 sgRNA) and Non-target controls (NTC) (Representative image, n = 3). Mean ± SEM, **p < 0.01

Although several studies have successfully used the CRISPR Synergistic Activation Mediator system to induce robust transcriptional activation of genes of interest, to our knowledge, the use of CRISPR activation has thus far not been used with the goal of altering EV cargo. In order to evaluate whether CRISPR can induce transcriptional activation of TSG-6 and induce downstream effects of EV contents, we first transduced an immortalized MSC line with the CRISPRa SAM system. After using antibiotic selection on the transduced cells, we selected an MSC line that was transduced with the entire CRISPR-SAM system with TSG-6 targeting sgRNAs (TSG-6 sgRNA), and an MSC line transduced with the CRISPR-SAM system and non-target control sgRNAs (NTC). We next assessed changes in TSG-6 gene expression in the selected MSC lines through RT-qPCR. The activation of TSG-6 was confirmed through a significant increase in TSG-6 expression (883.4 ± 134.0 fold change, p = 0.0027) in the “TSG-6 sgRNA” MSC line relative to non-target controls (Fig. 3A). Western blot analysis of MSC lysates revealed higher protein expression of TSG-6 in the “TSG-6 sgRNA” line than in NTC (Fig. 3B). Next, we measured the TSG-6 protein contents on harvested EV fractions, and observed increased levels of TSG-6 in EV samples from the CRISPR activated MSC line relative to EVs harvested from NTC (Fig. 3C). These experiments confirm the generation of a cell line overexpressing TSG-6. Furthermore, the observed overexpression on the gene and protein level of the MSCs was transmitted into the TSG-6 protein contents inside MSC-EVs.

**Fig. 4:**
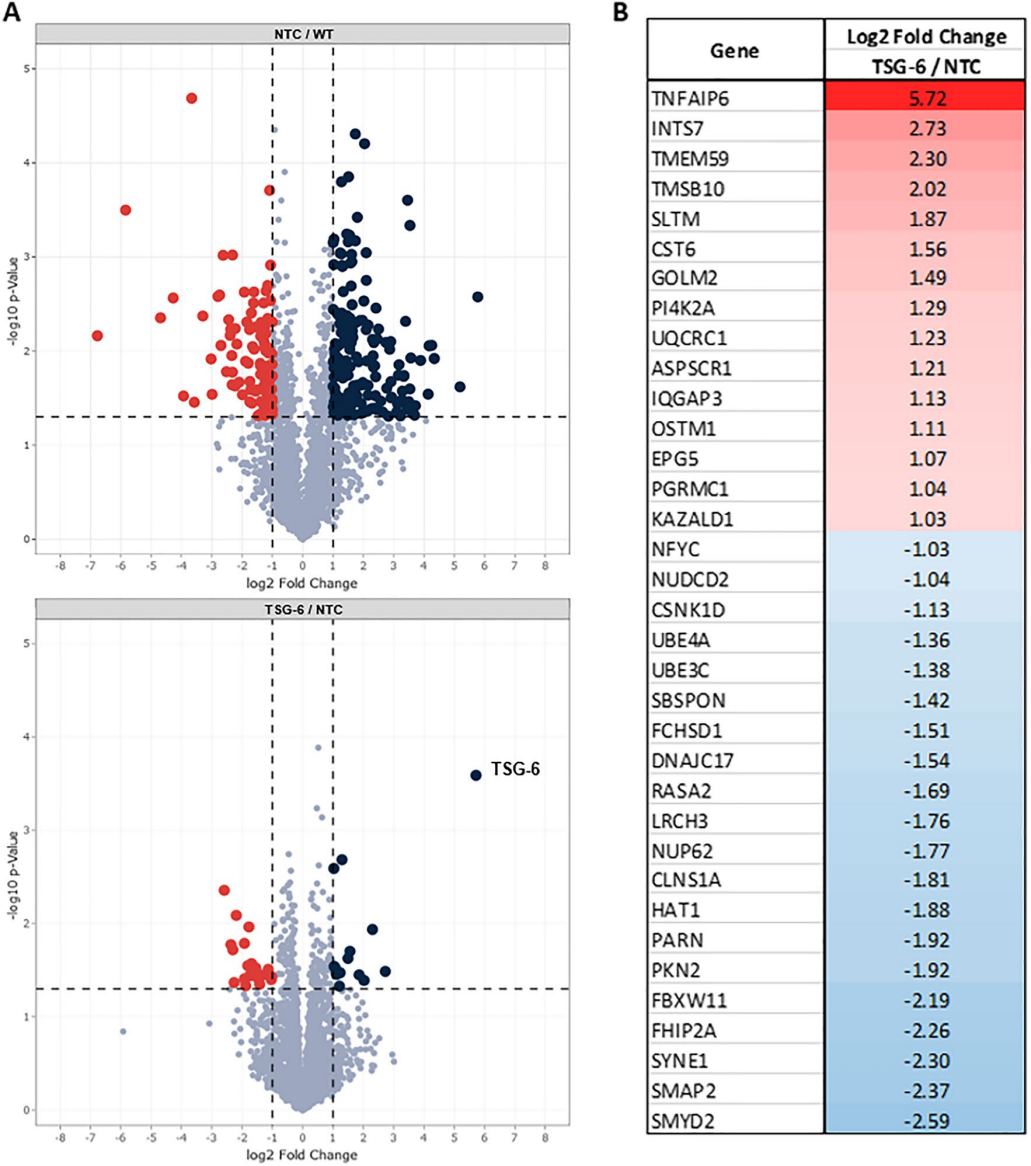
EV cargo proteomics. **A** Volcano plot representation displaying the change in EV protein abundance between NTC and WT groups (**A**, upper), and TSG-6 activated cells (TSG-6) and NTC groups (**A**, lower) (n = 3). Each dot represents a single protein quantified in the two conditions with the x-axis corresponding to the log2 fold change and y-axis corresponding to the log10 p-value from a student’s t-test. Significantly more abundant proteins in EVs (log2 fold change > 1, and p-value < 0.05) are represented on the top-right quarter (Blue), and significantly less abundant proteins in EVs (log2 fold change < − 1 and p-value < 0.05) are in the top-left quarter (red). The protein TSG-6 is represented by the top-right-most dot (log2 fold change = 5.72; p = 0.00025) in the TSG-6/NTC comparison. Significant proteomic differences were also observed between TSG-6 EVs and WT EVs (Supplementary Figure 2). **B** List of proteins with significantly upregulated or downregulated expression (TSG-6/NTC EV cargos). The complete list of analyzed proteins and their values is included in Supplementary Spreadsheet 1

To further investigate downstream effects from TSG-6 activation on EV cargo, we performed quantitative proteomics on EVs derived from TSG-6 activated MSCs (TSG-6), NTC, and wild-type controls (n = 3 for each group). A total of 3655 proteins were identified and quantified in the samples, and the relative protein abundances of the analyzed samples were analyzed (Supplementary Spreadsheet 1). When comparing the NTC and WT samples, 187 (5.12%) and 222 (6.07%) proteins exhibited significant (p < 0.05) log2 fold changes that were higher than 1, or smaller than − 1, respectively (Figure 4A). These results suggest that the EV proteome was significantly altered in response to the introduction of the CRISPR-SAM system and independent of the targeted gene. When controlling for these differences by comparing protein abundances between TSG-6 activated and NTC groups, only 15 (0.41%) proteins were significantly up-regulated (log2 fold change > 1 and p < 0.05) and 20 (0.54%) proteins were significantly down-regulated (log2 fold change < − 1 and p < 0.05). More specifically, protein TSG-6 showed the highest abundance difference, presenting a 5.72 log2 fold change relative to NTC (Fig. 4B).

We performed GO-term (GO-net), molecular pathway (Panther) and protein network (STRING) analyses to identify functional connections between the EV proteins for which significant differential abundances were observed (Supplementary Spreadsheet 2). For the comparison between NTC and WT samples, these analyses indicated a higher abundance of proteins associated e.g. with protein homeostasis and the immune system, which is in line with the larger proteomic changes observed in response to the CRISPR-SAM system and a potential stress response associated with these changes. However, no significant enrichment of GO-terms, pathways, or protein networks was found for EV proteins that showed differential abundance in TSG-6 vs NTC samples, suggesting that the identified EV proteins with altered abundances are not functionally related.

### IL-1β Stimulation Leads to the Expression of Pro-inflammatory Markers that can be Partially Reversed with TSG-6 Activated EVs

**Fig. 5:**
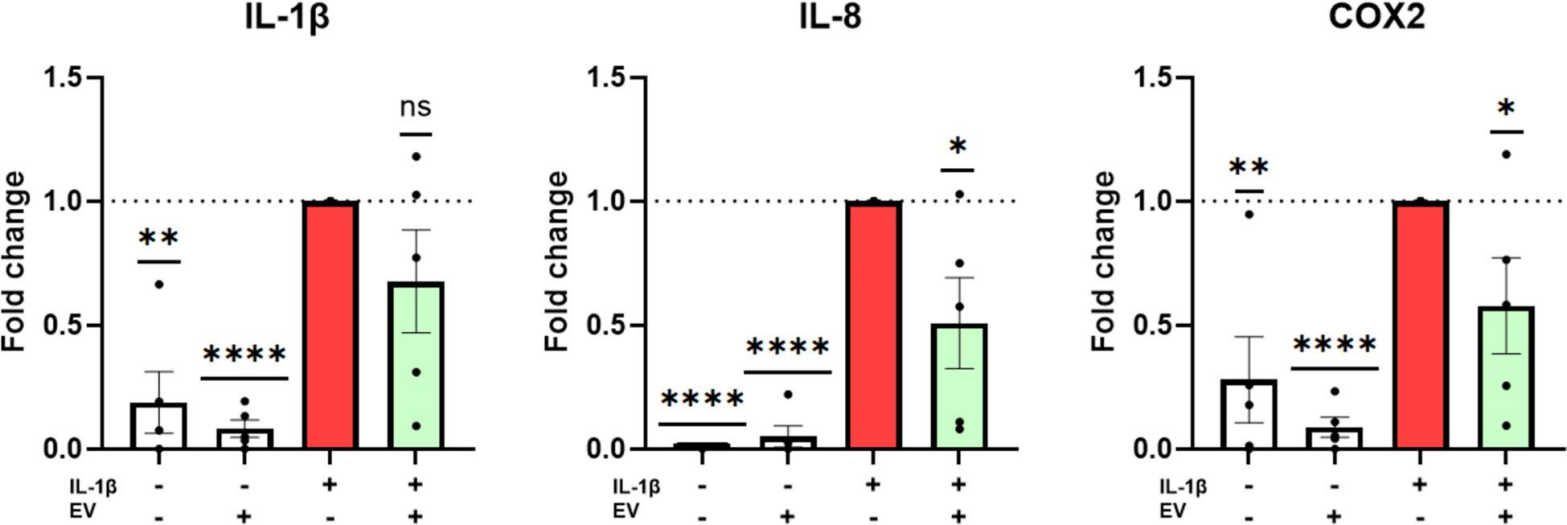
RT-qPCR data showing the effects of EV treatment of IVD cells pre-stimulated with IL-1β on the expression of inflammatory markers on different donors (n = 5). Mean ± SEM, *p < 0.05, **p < 0.01, ***p < 0.001, ****p < 0.0001 relative to IL-1β treatment (red bar)

It has been previously established that Interleukin-1 beta (IL-1β) induces the gene expression of pro-inflammatory and catabolic markers in IVD cell cultures, (e.g., IL-1β, IL-8, PTGS2, and others) by binding to its surface receptors and activating the NF-κB and MAPK signaling pathways. Therefore, in order to understand how the treatment with TSG-6 activated MSC-EVs can affect the inflammatory phenotype of degenerative IVDs, we performed a co-treatment with IL-1β and “TSG-6 activated” EVs. Gene expression of IL-1β, IL-8 and COX-2 was significantly upregulated upon IL-1β treatment relative to untreated controls (Fig. 5, white vs red bar). IL-1β, IL-8 and COX-2 showed partial downregulation in IL-1β + EV co-treatments relative to IL-1β treatment controls (Fig. 5, red vs green bar). In the case of IL-8 and COX-2 the downregulation was significant, with 0.51 ± 0.18 fold change, and 0.58 ± 0.19 fold change, respectively. In a small experimental sample, we furthermore compared the use of TSG-6 and NTC EV co-treatment of IVD cells pre-stimulated with IL-1β, but no statistically significant difference was observed between both treatment groups (Supplementary Figure 3).

## Discussion

Accumulating evidence suggests that EVs play a crucial role in the therapeutic effects of MSCs across various tissues, including the IVD [36, 63–66]. In this study, we applied CRISPR-Cas9 genomic engineering to modify MSC-derived EVs, aiming to enhance their therapeutic potential. As a proof-of-concept, we used CRISPR activation to overexpress TSG-6, a gene believed to drive several therapeutic benefits of MSCs [48–50]. Our key finding is that CRISPR-Cas9 activation significantly increased the concentration of TSG-6 in MSC-derived EVs while preserving their typical size distribution, morphology, and marker expression. Furthermore, the CRISPR-activated MSCs retained their trilineage differentiation capacity, comparable to unmodified MSCs. Despite the promising changes in EV cargo, including elevated TSG-6 (and other proteins) and reduced inflammatory marker expression in human IVD cells (with some donor-to-donor variability), the therapeutic efficacy of activated EVs did not surpass that of native EVs.

In this study, ASC52telo cells were chosen for EV production due to the limitations of primary MSCs, such as restricted number of passages and donor-to-donor variability. Establishing a genetically engineered cell line and sufficient cell expansion further complicate the use of primary cells. Moreover, variability in transcriptional activation responses across primary donors makes an immortalized MSC line, like ASC52telo, preferable for such studies [67–71]. ASC52telo cells have been thoroughly characterized and are widely utilized in research, including studies focused on MSC-derived EVs [72, 73]. For instance, Hindle et al. demonstrated large-scale production of ASC52telo-derived EVs for retinal pigment epithelial cell treatment, with therapeutic effects similar to those from primary cells [71]. Similarly, Katz et al. successfully used lentiviral vectors to overexpress SOX9 in ASC52telo cells, promoting chondrogenic properties [74]. In our study, immunofluorescence staining confirmed that TSG-6-activated ASC52telo cells maintained their stem cell properties, showing similar expression of adipogenic, osteogenic, and chondrogenic markers compared to unmodified ASC52telo cells. These results suggest that ASC52telo is a robust choice of cell line for CRISPR-SAM transcriptional activation and MSC-EV production. Of note, EVs from activated ASC52telo retained common EV characteristics, including morphology, size distribution, and marker expression.

Traditional gene overexpression methods often require introducing full-length transgenes into the host cell, which is cumbersome for multiplexing. In contrast, the dCas9-Activator approach is more efficient, targeting endogenous genes via short guide RNAs rather than transgenes, making it ideal for simultaneous gene activation [75–77]. We employed the SAM CRISPR Activator Lentivirus system, widely used for robust transcriptional activation [43, 78, 79]. Our results revealed an over 800-fold increase in TSG-6 mRNA in activated cells compared to controls, with a significant increase in TSG-6 protein in both, MSC lysates and MSC-derived EVs. Proteomic analysis of the EVs showed altered protein levels, with 35 proteins significantly affected. While TSG-6 overexpression likely drove some changes, the exact mechanisms remain unclear, as no direct functional connections between the altered proteins were identified.

Among the list of identified proteins, various cargo components with potential therapeutic relevance were detected. In addition to TSG-6, numerous interesting cytokines (CCL2, CXCL8, CSF1), growth factors (VEGF, HGF, TGFB1-3, PDGFA), and other proteins of biological interest (such as metalloproteinase inhibitors TIMP1 and TIMP2), were found. The therapeutic effect of EVs is likely in part consequence of the EV proteome, and as such, more in depth analysis of EV content will be required in the future. The full list of quantified proteins is included in Supplementary Spreadsheet 1.

Our findings confirm the efficacy of this approach for target gene (i.e. TSG-6) overexpression and its reflection in EV content. It should however be noted that NTC and WT MSC-EVs showed differences in EV content, which may be related to a stress response to the CRISPR-SAM system or the lentiviral transduction. Future research will focus on the simultaneous overexpression of multiple genes to further explore and expand the applications of this approach.

For our proof-of-concept MSC-EV treatment, we used an *in vitro* IL-1β-induced inflammatory IVD cell model, which mimics degenerative processes via the NF-κB and MAPK pathways [9, 14]. By inducing an early inflammatory and degenerative phenotype in IVD cells with IL-1β, we could evaluate the therapeutic potential of the EVs by monitoring gene expression changes. To minimize interference from exogenous factors, we employed serum-free media for both EV production and IVD treatments. Serum-free harvest of EVs is important to avoid interference from fetal bovine serum (FBS) components, including EVs and growth factors. Although some researchers use EV-depleted serum for EV harvesting, non-EV components in the serum may still affect the results, further supporting the use of serum-free culture.

EV dosage was standardized by particle number, as determined by NTA, relative to the number of treated cells (1000 particles per IVD cell). Similarly, the number of seeded MSCs for EV production was kept constant. This approach, combined with the use of the same MSC line within a limited passage range, was intended to minimize variability. However, despite these efforts, we did not observe statistical significance in the treatment efficacy of “activated EVs” compared to that of NTC EVs. Nonetheless, both EVs types showed promising anti-inflammatory capacities, whereby some donors had a more robust response to the EV treatment than others. Donor variability, possibly arising from variations in donor age, degree of tissue degeneration, or other factors, is a well-known challenge in IVD research [80]. Future studies based on this model will include additional EV-depleted controls obtained from the supernatant of the EV harvest (to test for functional effects induced by co-isolated elements of the conditioned media), will be conducted on a larger sample set (to potentially identify donor characteristics that affect responsiveness), and treatment durations will be extended to assess long-term effects of EV treatment. We also plan to further explore the altered EV content by analyzing the transcriptomic profile, including total RNA and small RNAs. Additionally, the effects of MSC-EVs on macrophage polarization will be investigated to evaluate their immunomodulatory potential. Investigating macrophages is of particular interested as their presence and contribution to an inflammatory environment has been demonstrated in degenerated IVDs [7, 81, 82]. Previous studies suggest that MSC-EVs polarize macrophages toward an M2 phenotype, with TSG-6 playing a role in this transition [63, 83, 84]. Along with immunomodulatory gene targets, future work will also focus on boosting EV biogenesis by targeting genes involved in EV production pathways.

In conclusion, this study demonstrates the feasibility of using CRISPR tools to modify EV content. Our CRISPR-activated MSC line overexpresses TSG-6, resulting in altered EV cargo. These findings represent a significant advancement in the development of MSC-EV-based therapies for IVD degeneration and other degenerative and inflammatory conditions.

## Supplementary Information

Below is the link to the electronic supplementary material.

Supplementary file1 (DOCX 1569 KB)

Supplementary file2 (XLSX 3015 KB)

Supplementary file3 (XLSX 16 KB)

## Data Availability

The mass spectrometry proteomics data have been deposited to the ProteomeXchange Consortium via the PRIDE [85] partner repository with the dataset identifier PXD060381. All other datasets are available in the Figshare repository (10.6084/m9.figshare.27270243.v1).
