# Supplementary material for: CRISPR-dCas9 Activation of TSG-6 in MSCs Modulates the Cargo of MSC-Derived Extracellular Vesicles and Attenuates Inflammatory Responses in Human Intervertebral Disc Cells In Vitro
Source: Cell Mol Bioeng. 2025 Feb 5;18(1):83–98. doi: 10.1007/s12195-025-00843-4 (PMC11813855; doi:10.1007/s12195-025-00843-4)
Supplement: Supplementary file 1 — Supplementary file1 (DOCX 1569 KB) [file 12195_2025_843_MOESM1_ESM.docx]

Supporting Information

**Supplementary Table 1**: Patient characteristics of surgically exised degenerated IVD biopsies. f = female; m = male; DDD = degenerative disc disease; L = lumbar; C = cervical; S = sacrum.

| **Donor** | **Age** | **Sex** | **Diagnosis** | **Disc Level** | **Pfirrmann Grade** |
| --- | --- | --- | --- | --- | --- |
| 1 | 40 | m | Herniation | L4-5 | 3 |
| 2 | 29 | f | Herniation | L4-5 | 3 |
| 3 | 65 | f | Herniation | C6-C7 | 4 |
| 4 | 41 | m | DDD | L5-S1 | 3 |
| 5 | 47 | f | DDD | L4-5 | 4 |


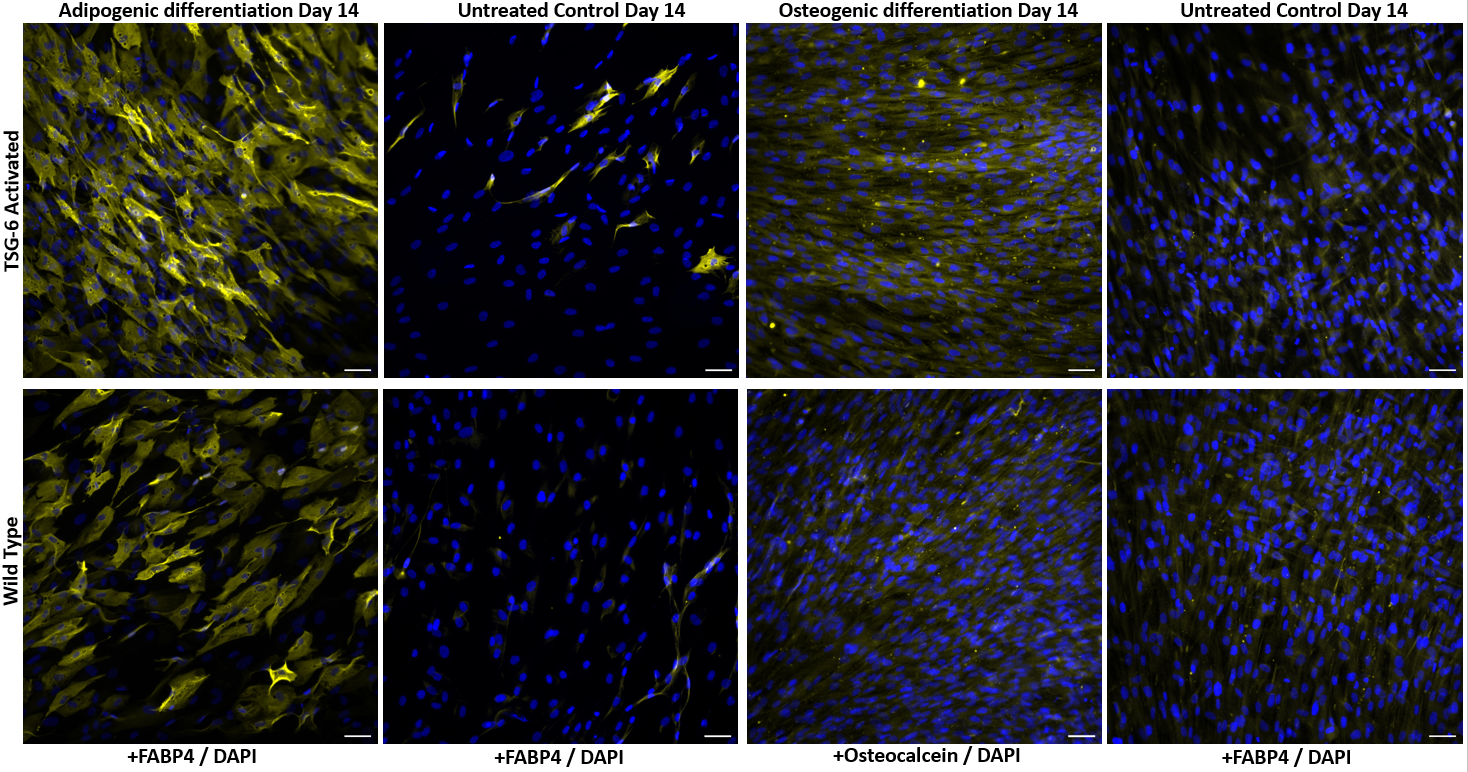


**Supplementary Figure 1**: ICC staining of Adipogenic and Osteogenic differentiation of unmodified (= wild type) MSCs and TSG-6-actived MSCs, compared to untreated controls. Adipogenic -FABP4 [Yellow], and osteogenic - Osteocalcein [Yellow]. Cell nuclei were stained with DAPI (blue). Scale bar 50 µm. (n =1).


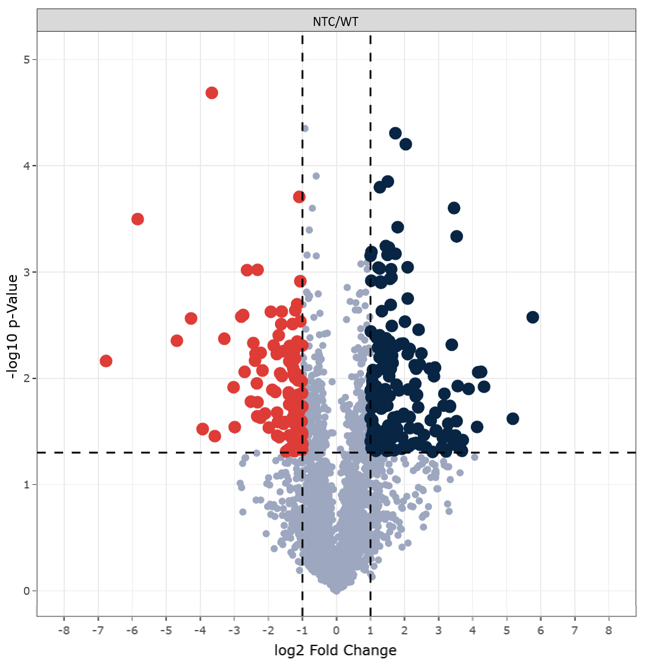


**Supplementary Figure 2:** Volcano plot representation displaying the change in protein abundance between Non-Target control (NTC) and wild type (WT) groups (n = 3). Each dot represents a single protein quantified in the two conditions with the x-axis corresponding to the log2 fold change and y-axis corresponding to the log10 p-value from a student’s t-test. Significantly more abundant proteins (log2 fold change > 1, and p-value < 0.05), are represented on the top-right quarter (Blue), and significantly less abundant (log2 fold change < -1 and p-value < 0.05) proteins are in the top-left quarter (red).


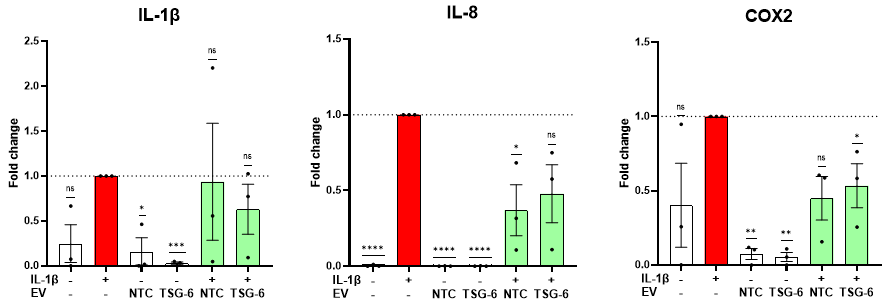


**Supplementary Figure 3:** RT-qPCR data showing the comparison between NTC EVs and TSG-6 EVs co-treatment of IVD cells pre-stimulated with IL-1β (n = 3). Mean ± SEM, * p < 0.05, ** p < 0.01, **** p < 0.0001 relative to IL-1β treatment [red bar] No significant differences were observed between NTC EVs and TSG-6 EVs [i.e. between the two green bars].
